# Functional characterization of ivermectin binding sites in α1β2γ2L GABA(A) receptors

**DOI:** 10.3389/fnmol.2015.00055

**Published:** 2015-09-25

**Authors:** Argel Estrada-Mondragon, Joseph W. Lynch

**Affiliations:** ^1^Queensland Brain Institute, The University of QueenslandBrisbane, QLD, Australia; ^2^School of Biomedical Sciences, The University of QueenslandBrisbane, QLD, Australia

**Keywords:** Cys-loop receptor, ligand-gated, ion channel, avermectin, macrocyclic lactone, GABA, pharmacology

## Abstract

GABA_A_ receptors (GABA_A_Rs) are the major inhibitory neurotransmitter receptors in the brain and are therapeutic targets for many indications including sedation, anesthesia and anxiolysis. There is, however, considerable scope for the development of new therapeutics with improved beneficial effects and reduced side-effect profiles. The anthelminthic drug, ivermectin, activates the GABA_A_R although its binding site is not known. The molecular site of action of ivermectin has, however, been defined by crystallography in the homologous glutamate-gated chloride channel. Resolving the molecular mechanisms of ivermectin binding to α1β2γ2L GABA_A_Rs may provide insights into the design of improved therapeutics. Given that ivermectin binds to subunit interfaces, we sought to define (1) which subunit interface sites it binds to, (2) whether these sites are equivalent in terms of ivermectin sensitivity or efficacy, and (3) how many must be occupied for maximal efficacy. Our approach involved precluding ivermectin from binding to particular interfaces by introducing bulky M3 domain 36′F sidechains to the “+” side of those interfaces. We thereby demonstrated that ivermectin produces irreversible channel activation only when it binds to the single γ2L-β2 interface site. When it binds to α1-β2 sites it elicits potentiation of GABA-gated currents but has no irreversible activating effect. Ivermectin cannot bind to the β2-α1 interface site due to its endogenous bulky 36′ methionine. Replacing this with an alanine creates a functional site at this interface, but surprisingly it is inhibitory. Molecular docking simulations reveal that the γ2L-β2 interface forms more contacts with ivermectin than the other interfaces, possibly explaining why ivermectin appears to bind irreversibly at this interface. This study demonstrates unexpectedly stark pharmacological differences among GABA_A_R ivermectin binding sites.

## Introduction

Receptors of the pentameric ligand-gated ion channel (pLGIC) family mediate fast synaptic neurotransmission in the nervous system. In vertebrates, the pLGIC family includes the anion-permeable glycine receptor (GlyR) and GABA type-A receptor (GABA_A_R) and the cation-permeable nicotinic acetycholine receptor (nAChR) and serotonin type-3 receptor (5-HT_3_R). Invertebrate species are known to express a variety of other pLGIC receptor subtypes, including a glutamate-gated chloride channel receptor (GluClR). pLGICs comprise five subunits arranged in a ring to form a central water-filled pore that spans the cell membrane. Each subunit can be divided into three functional domains. The extracellular domain contains the binding sites for the neurotransmitter agonists. The transmembrane domain comprises twenty α-helices (four per subunit) arranged in concentric layers around a central aqueous pore, with M2 directly lining the permeation pathway, M1 and M3 shielding M2 from the surrounding lipid bilayer, and M4 being the outermost segment. As detailed below, the transmembrane region provides the binding sites for hydrophobic ligands such as alcohols, anesthetics and macrocyclic lactones such as ivermectin. The third receptor domain is intracellular and contains phosphorylation sites and binding sites for synaptic clustering proteins.

Most members of the human pLGIC family are targeted by drugs of major therapeutic importance. GABA_A_Rs, for example, are established therapeutic targets for alcohol withdrawal, muscle relaxation, sedation, anaesthetia, seizure control and anxiolysis, although there is considerable scope for the development of new therapeutics with improved beneficial effects and a reduced propensity for dependency and other side-effects.

The gold standard anthelmintic drug, ivermectin, is widely used for controlling filarial and arthropod parasitic infestations in humans, livestock and agriculture (Crump and Omura, 2011; Campbell, 2012). Its biological target is the GluClR, a pLGIC that is highly expressed in nerve and muscle cells of nematodes and arthropods, but absent in vertebrate species. Low nanomolar concentrations of ivermectin irreversibly activate a chloride influx through these GluClRs that electrically silences nerve and muscle activity, leading to death by flaccid paralysis or starvation (Wolstenholme and Rogers, 2005). Importantly, ivermectin and related compounds also bind to and either directly activate or positively modulate several vertebrate pLGICs including GlyRs (Shan et al., 2001; Lynagh and Lynch, 2010; Lynagh et al., 2011), GABA_A_Rs (Sigel and Baur, 1987; Krüsek and Zemková, 1994; Adelsberger et al., 2000), and α7 nAChRs (Krause et al., 1998), albeit at relatively low potencies. Ivermectin also activates the structurally-unrelated P2X_4_ cation channel (Khakh et al., 1999). A 3.3 Å crystal structure of ivermectin docked to the *C. elegans* α GluClR has recently been published (Hibbs and Gouaux, 2011; Althoff et al., 2014). Although this structure clearly defines the orientation of ivermectin in its site, a functional analysis of ivermectin-receptor interactions in the human GlyR and amino acid sequence comparisons of ivermectin-sensitive and ivermectin-insensitive pLGICs from various phyla suggest that the molecular interactions mediating ivermectin binding remain to be delineated (Lynagh and Lynch, 2012a,b).

Understanding the molecular interactions between ivermectin and its binding sites on the GABA_A_R may define new therapeutic pharmacophores that could be useful in the design of improved treatments for neurological disorders such as those listed above. The most abundant GABA_A_R subtype in the brain is formed from α1, β2, and γ2L subunits in a β2-α1-β2-α1-γ2L stoichiometry in an anticlockwise orientation when viewed toward the membrane from the extracellular membrane side (Sieghart and Sperk, 2002; Gallagher et al., 2004; Olsen and Sieghart, 2009). Given that ivermectin binds at subunit interfaces (Lynagh and Lynch, 2012a), this stoichiometry implies four structurally distinct sites per receptor. Although we know that ivermectin activates these receptors (Adelsberger et al., 2000), we do not know (1) which subunit interface sites it binds to, (2) whether these structurally distinct sites are equivalent in terms of ivermectin sensitivity or efficacy, or (3) how many of them must be occupied for maximal ivermectin efficacy. In addition, it has been shown that large sidechains at the 36′ position in the M3 domain block ivermectin access to its site in the α1 GlyR and α3β GluClR (Lynagh and Lynch, 2010; Lynagh et al., 2011). It is currently unclear whether this also applies to the GABA_A_R, and resolving this would help confirm whether ivermectin binds to GABA_A_Rs in the same orientation as it binds to GluClRs. Here we sought to address all these questions by investigating heterologously expressed human α1β2γ2L GABA_A_Rs that incorporate site-directed mutations to 36′ residues with the aim of constraining ivermectin binding to defined interfaces. The effects of ivermectin on mutant receptors are quantitated via patch clamp electrophysiology and the results are interpreted with the aid of molecular structural modeling and computational ligand docking.

## Materials and methods

### Chemicals

Ivermectin and GABA were obtained from Sigma-Aldrich. Stocks of 10 mM ivermectin were dissolved in dimethyl sulfoxide and stored at −20°C. GABA was maintained as a 100 mM stock in water.

### Molecular biology

The human α1, β2, and γ2L GABA subunit cDNAs were independently subcloned into the pcDNA3.1 plasmid vector (Life Technologies, Waltham, MA). Site-directed mutagenesis was done using the QuikChange mutagenesis kit (Agilent, Santa Clara, CA). Successful incorporation of all the mutants was confirmed by DNA sequencing.

### HEK-293 cell culture and transfection

HEK-293 cells were cultured in Dulbecco's modified Eagle's medium (Life Technolgies, Waltham, MA) containing penicillin/streptomycin (Sigma-Aldrich, St. Luis, MO) and Fetal Bovine Serum (HyClone, Logan, UT), and split onto glass coverslips in 60 mm dishes. On the next day, cells were transiently transfected with the GABA_A_R cDNAs of interest (at a plasmid transfection ratio of 1α1:1β2:3γ2L) and an empty pEGFP vector (Clontech, Mountainview, CA) as a fluorescent transfection marker via a calcium phosphate method.

### Patch clamp electrophysiology

Transfected cells on glass coverslips showing GFP fluorescence were used in experiments. Patch clamp pipettes were pulled from borosilicate glass capillary tubes (Hirschmann Laborgeräte, Eberstadt, Germany) using a horizontal pipette puller (P97, Sutter Instruments, Novato, CA) and had tip resistances of 1.5–3.5 MΩ when filled with intracellular solution, consisting of (in mM): 145 CsCl, 2 CaCl_2_, 2 MgCl_2_, 10 HEPES, and 5 EGTA, adjusted to pH 7.4 with CsOH. Extracellular solution consisted of (in mM): 140 NaCl, 5 KCl, 2 CaCl2, 1 MgCl2, 10 HEPES, and 10 D-glucose, adjusted to pH 7.4 with 2M NaOH. Cells were voltage-clamped at −70 mV in the whole-cell recording configuration and membrane currents were recorded using Axon Multiclamp 700B amplifier and pClamp 10 software (Molecular Devices, Sunnyvale, CA). Membrane currents were filtered at 500 Hz and digitized at 2 kHz. Solutions for experiments were prepared from stocks on the day of recording. Solutions were applied to cells via a purpose-built gravity-fed perfusion system fabricated from polyethylene tubing. Experiments were conducted at room temperature (19–22°C). Applying increasing concentrations of drugs to cells generated GABA, and ivermectin concentration-response relations as outlined below.

### Molecular modeling and docking

The crystal structure of the *C. elegans* α GluClR in the wide-open pore conformation in complex with ivermectin and glutamate (PDB entry 3RIF; Hibbs and Gouaux, 2011), was used as a template for the homology modeling of the human GABA subunits α1, β2, and γ2L, as well as the Single-Point Mutants: α1^A36′F^, β2^M36′A^, and γ2L^S36′F^. The sequence of the human GABA_A_R subunits α1 (Uniprot entry P14867), β2 (P47870), and γ2L (P18507) were aligned with the GluClR sequence using ClustalW. Each GABA_A_R subunit modeling was performed with Modeler 9.10 (Sali and Blundell, 1993). One hundred models of each subunit were generated and ranked according to their DOPE scores. The respective WT or mutant α1 and β2 subunits were projected twofold and the γ2L subunit one-fold, using the coordinates of GluClR in order to assemble the respective pentamer. Each model with the lowest score was selected and was checked with the “What if” (swift.cmbi.ru.nl), and MolProbity (smb.slac.stanford.edu) protein structure validation servers. For the docking, the PDB was prepared as a “ICM object” using the ICM Pocket Finder method in the Internal Coordinate Mechanics software (ICM-Pro Molsoft LLC, San Diego, CA), identifying three common binding pockets in the α1-β2, α1-γ2L, γ2L-β2, and the mutant β2^M36′A^-α interfaces: one pocket in the extracellular domain and two in the transmembrane domains of the model. One of the transmembrane domain pockets corresponded with the Ivermectin Binding Site reported for the GluCl receptor, this pocket was used for all the following tests. Ivermectin was loaded from the ChemSpider database (ID 16736314) and was docked in the assigned pocket interface using the flexible docking ICM-biased probability Monte Carlo (BPMC) method. Three independent docking runs were performed for each interface of the GABA_A_ receptor model with the length of the docking simulation adjusted by the default thoroughness value (thoroughness = 1). The docking poses were selected on the basis of their docking scores and if appropriate the capacity of forming hydrogen bonds between the ligand and the receptor. The program predicted similar binding poses for the most sites of each interface with the molecule of IVM, the average difference between the highest and the lowest Root Mean Square Deviation (RMSD) predictions for all of the interacting residues that are common to all four interface binding sites was ~2 Angstrom. Each molecule of ivermectin docked was manually inspected in its docked pose for receptor residues in each interface making contact lower than a spatial chemical distance of 5 Å^2^ using the contact area function of the ICM-Pro software, this distance satisfices the requirements for the formation of hydrogen bonds, disulfide bonds, salt bridges and hydrophobic interactions. The ligand-protein model complex was saved in PDB format and images were rendered using ICM-Pro software and the Pymol Molecular Graphics System, Version 1.3 (Schrodinger, 2010).

### Statistical analysis

GABA and ivermectin concentration-response experiments were performed as described below. For each GABA concentration-response experiment, the half-maximal agonist concentration (EC_50_), Hill coefficient (n_H_) and saturating current magnitude (I_max_) values were determined by fitting individual concentration-response relationships with the 3-parameter Hill equation (Prism 6.0, GraphPad Software Inc., San Diego, CA, USA). Results are expressed as mean ± S.E.M from at least 3 experiments. Ivermectin concentration-response relations were normalized relative to the GABA-activated I_max_ in the same cell and then expressed as mean ± S.E.M from 3 to 6 experiments. Ivermectin concentration-response relations were fitted with cubic spine curves. In all experiments, ANOVAs or *t*-tests, as appropriate, were used to compare indicated values with *P* < 0.05 representing significance.

## Results

### Effects of ivermectin on wild type α1β2γ2L GABA_A_Rs

**Figure 1:**
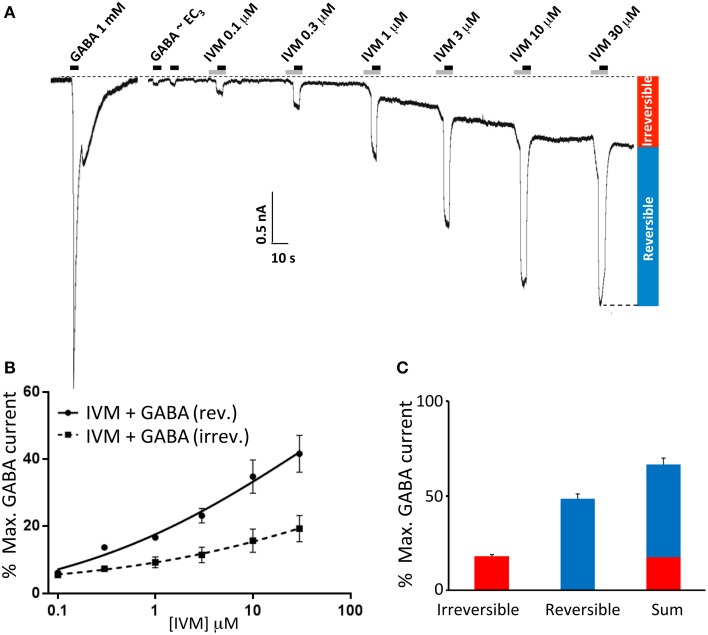
**Ivermectin modulation of wild type α1β2γ2L GABA_A_Rs**. In this and subsequent figures, all displayed traces were recorded from HEK293 cells expressing WT or mutated α1β2γ2L receptors using whole cell recording, and the durations of ivermectin and GABA applications are indicated by gray and black bars, respectively. **(A)** Sample recording showing the effect of repeated applications of EC_3_ GABA together with increasing concentrations of ivermectin at 1 min intervals as indicated. A current response to saturating GABA from the same cell is also shown. **(B)** Averaged ivermectin concentration-response relationships from 4 to 5 cells, with error bars shown as SEM. Ivermectin-induced reversible and irreversible currents, measured as shown in **(B)**, are plotted separately as circles and squares, respectively. **(C)** Mean saturating magnitudes of reversible and irreversible ivermectin-modulated currents (defined as indicated in **A**), and their sum, plotted as a percentage of the saturating GABA-activated current. Mean GABA EC_50_, n_H_ and I_max_ values for this and all other constructs are provided in Table 1.

Ivermectin concentration-response relationships were determined by applying ivermectin at progressively increasing concentrations (0.1, 0.3, 1, 3, 10, and 30 μM) for 10 s periods at 1 min intervals. An EC_3_ concentration of GABA was co-applied with ivermectin for the second 5 s period of every 10 s application. A sample recording from α1β2γ2L GABA_A_Rs using this protocol is shown in Figure 1A. Under these conditions, ivermectin induced both a potentiation of the reversible GABA-gated current plus an irreversibly-activated current component. Mean concentration-response relationships for both responses were normalized to the saturating GABA-gated current in the same cell and plotted separately (Figure 1B). In addition, the mean saturating magnitudes of the reversible and irreversible current components relative to the saturating GABA-gated current magnitude in the same cell are plotted in Figure 1C. Throughout the rest of this study we quantitated the effects of ivermectin as illustrated in Figure 1B.

**Table 1:** **Concentration-response curve fit parameters for GABA at wild type and mutant GABA_A_Rs**.

**GABA receptor**	**GABA EC_50_ (μM)**	**n_H_**	**I_max_ (nA)**	**n**
α1β2γ2L (wild type)	0.92±0.19	1.4±0.2	3.2±0.6	5
α1^A36′F^ β2γ2L	0.13±0.01	2.1±0.1	3.5±1.5	4
α1β2γ2L^S36′F^	4.42±0.17^***^	1.4±0.1	3.6±1.1	4
α1^A36′F^ β2γ2L^S36′F^	4.38±0.12^***^	1.5±0.1	3.8±1.3	5
α1β2	0.80±0.03	1.1±0.2	1.8±0.5	4
α1^A36′F^ β2	0.13±0.01	2.1±0.1	2.5±0.5	4
α1β2^M36′A^γ2L	0.76±0.02	0.9±0.1	2.9±0.6	5
α1^*A*36′*F*^β2^M36′A^γ2L^S36′F^	2.62±0.03^***^	0.9±0.1	3.0±0.6	4

*** *P < 0.001 using One-Way ANOVA Dunnett's with multiple comparisons test*.

### Control of ivermectin binding to subunit interface sites

**Figure 2:**
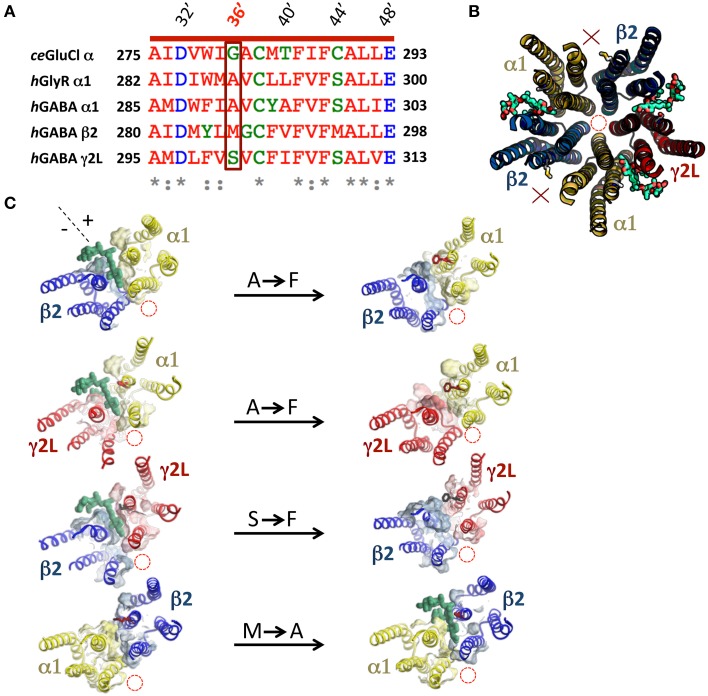
**Molecular modeling of ivermectin binding to its subunit interface binding sites in the α1β2γ2L GABA_A_R**. **(A)** Amino acid sequence alignment of M3 residues of the human α1, β2, and γ2L GABA_A_R subunits, the human α1 GlyR subunit and the *C. elegans* α GluClR subunit with their respective 36′ residues highlighted in a box. **(B)** Molecular structural model of the transmembrane domains of a wild type β2-α1-β2-α1-γ2L GABA_A_Rviewed from the extracellular side of the membrane along the pore axis. This receptor accommodates three ivermectin molecules (green) in their subunit interface binding sites. In this and subsequent model structures, the pore location is denoted by the red dashed circle, 36′ sidechains and bound ivermectin molecules (green) are shown in stick form and interfaces that do not bind ivermectin are indicated by a red X. **(C)** This panel shows the effect of mutagenesis on the ability of ivermectin to bind at individual interfaces. The left column displays single subunit interfaces formed from (top to bottom) α1-β2, α1-γ2L, γ2L-β2, and β2-α1 interfaces, with ivermectin docked where possible. The right column shows the same four subunit interfaces incorporating the indicated mutations (A36′F, S36′F, or M36′A).

A previous study showed that the volume of the M3 residue at 36′ position (numbered according to the standard M2 domain residue numbering system) is a crucial determinant of ivermectin sensitivity in both the human α1 GlyR and the *H. contortus* α3β GluClR (Lynagh and Lynch, 2010). In both receptors, 36′G resulted in exquisite (low nanomolar) ivermectin sensitivity, 36′S and 36′A produced receptors with high nanomolar—low micromolar sensitivity, whereas larger residues (notably 36′F) eliminated ivermectin sensitivity entirely. As the 36′ sidechain lines the mouth of the ivermectin site on the “+” side of the subunit interface (Hibbs and Gouaux, 2011), it is likely that a large volume sidechain at this position sterically hinders ivermectin from entering its site (Lynagh et al., 2011; Lynagh and Lynch, 2012a). A sequence alignment of the α1, β2, and γ2L GABA_A_R subunits predicts that the α1 and γ2L subunits, which contain 36′A and 36′S, respectively, should support ivermectin binding sites but the β2 subunit, which contains a 36′M, should not (Figure 2A). In support of this, molecular modeling of ivermectin binding to the α1β2γ2L GABA_A_R reveals that it is able to dock into sites at the α1-β2, α1-γ2L, and γ2L-β2 interfaces but not at the β2-α1 interface (Figure 2B). In agreement with this, a recent study on an insect RDL homomeric GABA_A_R also found that the G36′M mutation eliminated ivermectin sensitivity (Nakao et al., 2015). We introduced the A36′F and S36′F mutations into the α1 and γ2L subunits, respectively, with the aim of eliminating existing ivermectin sites. We also introduced the M36′A mutation into the β2 subunit which we predicted would decrypt a possible site at the β2-α1 interface. According to our molecular modeling simulations, the A/S36′F mutations eliminated ivermectin binding whereas the M36′A mutations created an ivermectin site (Figure 2B). Of course, these predictions need to be validated by functional analysis.

**Figure 3:**
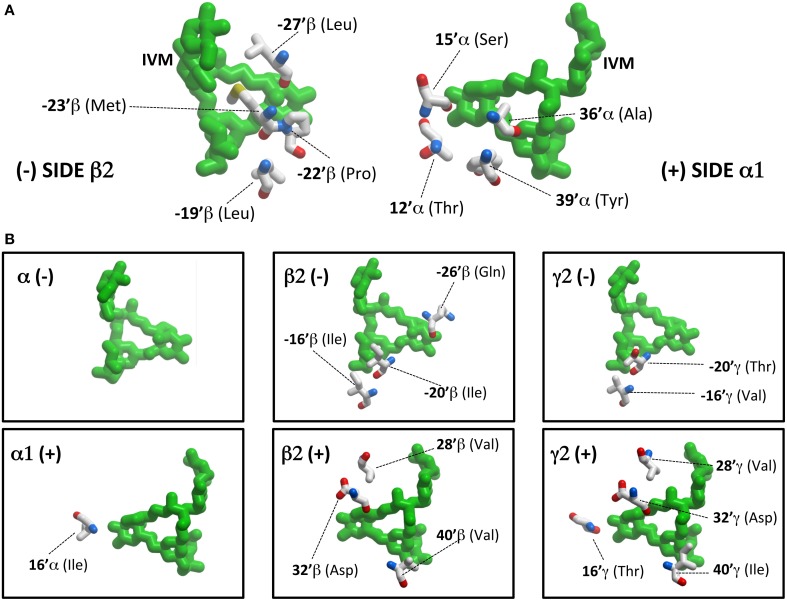
**Ivermectin contact residues**. **(A)** This panel shows ivermectin bound at the α1-β2 interface, viewed from within the plane of the membrane from the (−) side of the interface (left panel) and the (+) side of the interface (right panel). Note it only displays interacting residues that are common to all four interface binding sites. **(B)** This panel shows additional interacting residues, relevant to particular interfaces only. For example, at the β2 (−) interface, ivermectin interacts with a total of 7 residues (4 depicted in **A** and 3 depicted in **B**).

However, before presenting the functional analysis, we describe in more detail the molecular interactions predicted to exist at each interface site. Table 2 provides a summary of all transmembrane α1β2^M36′A^γ2L GABA_A_R residues predicted to interact with ivermectin in our model structure. Corresponding ivermectin binding residues from the *C. elegans* α GluClR crystal structure (Hibbs and Gouaux, 2011) are also displayed. Residues in gray are predicted not to bind ivermectin, whereas asterisked residues are predicted to form hydrogen bonds with ivermectin. The spatial orientations of these residues relative to docked ivermectin are depicted in Figure 3. Note that Figure 3A only displays those interacting residues that are common to all four interface binding sites. The additional interacting residues relevant to particular interfaces are shown in Figure 3B. According to Figure 3, the α1-β2 and α1-γ2L interfaces are structurally similar, with ivermectin-interacting residues sharing similar characteristics of polarity and hydrophobicity. On the other hand, the γ2L-β2 interface shows a greater number of residues contributing polar contacts that should result in the pocket forming tighter contacts around the bound ivermectin molecule. The decrypted β2-α1 interface has a similar number of contacts as the α1-β2 and α1-γ2L interfaces, although their spatial distribution patterns differ significantly (Figure 3B).

**Table 2:** **Transmembrane residues predicted to contribute to ivermectin sensitivity in the ***C. elegans*** α GluClR and the human α1β2^M36′A^γ2L GABA_A_R**.

	**α GluClR**	**α1 GABA_A_R**	**β2^M36′A^ GABA_A_R**	**γ2L GABA_A_R**
**M1 POSITION**				
−16′	Ile	Ile	**Ile** RMSD = 0.2 Å	**Val** RMSD = 0.2 Å
−19′	**Met**	**Met** RMSD = 0.4 Å	**Leu** RMSD = 0.1 Å	**Leu** RMSD = 0.6 Å
−20′	Cys	Ile	**Ile** RMSD = 0.1 Å	**Thr** RMSD = 0.6 Å
−22′	**Pro**	**Pro** RMSD = 0.2 Å	**Pro** RMSD = 0.1 Å	**Pro** RMSD = 0.1 Å
−23′	**Ile**	**Leu** RMSD = 0.1 Å	**Met** RMSD = 0.2 Å	**Ile** RMSD = 0.6 Å
−**26**	**Gln**	Gln	**Gln** RMSD = 0.1 Å	Gln
−27′	Leu^*^ 2.5 Å-C7OH	Ile^*^ 2.6 Å-C7OH RMSD = 0.1 Å	Leu^*^ 2.6 Å-C7OH RMSD = 0.1 Å	Ile^*^ 2.5 Å-C7OH RMSD = 0.5 Å
**M2 POSITION**				
12′	**Thr**	**Thr** RMSD = 0.1 Å	**Thr** RMSD = 0.1 Å	**Thr** RMSD = 0.1 Å
15′	Ser^*^ **2.6 Å-C5OH**	Ser^*^ RMSD = 0.1 Å **2.8 Å-C5OH**	**Asn** RMSD = 1.2 Å	Ser^*^ RMSD = 0.4 Å **2.8 Å-C5OH**
16′	Ala	**Ile** RMSD = 0.4 Å	Thr	**Thr** RMSD = 0.4 Å
**M3 POSITION**				
28′	**Ile**	Ala	**Val** RMSD = 0.8 Å	**Val** RMSD = 0.4 Å
32′	**Asp**	Asp	**Asp** RMSD = 1.5 Å	**Asp** RMSD = 1.6 Å
36′	**Gly**	**Ala** RMSD = 0.1 Å	**Ala** RMSD = 0.1 Å	**Ser** RMSD = 0.3 Å
39′	**Met**	**Tyr** RMSD = 0.4 Å	**Phe** RMSD = 0.7 Å	**Phe** RMSD = 0.9 Å
40′	**Thr**	Ala	**Val** RMSD = 0.5 Å	**Ile** RMSD = 0.3 Å

Residues in gray are predicted not to bind ivermectin. The number below each residue represents the Root Mean Square Deviation (RMSD) in residue position calculated from three independent docking simulations.

* *Residues forming H-bonds with hydroxyls attached to carbons C5 or C7 of the ivermectin molecule. The distances between the participating hydrogens are indicated. The values shown in bold are the distances in Angstroms between the residues forming H-bonds*.

### GABA concentration-response relationships

**Figure 4:**
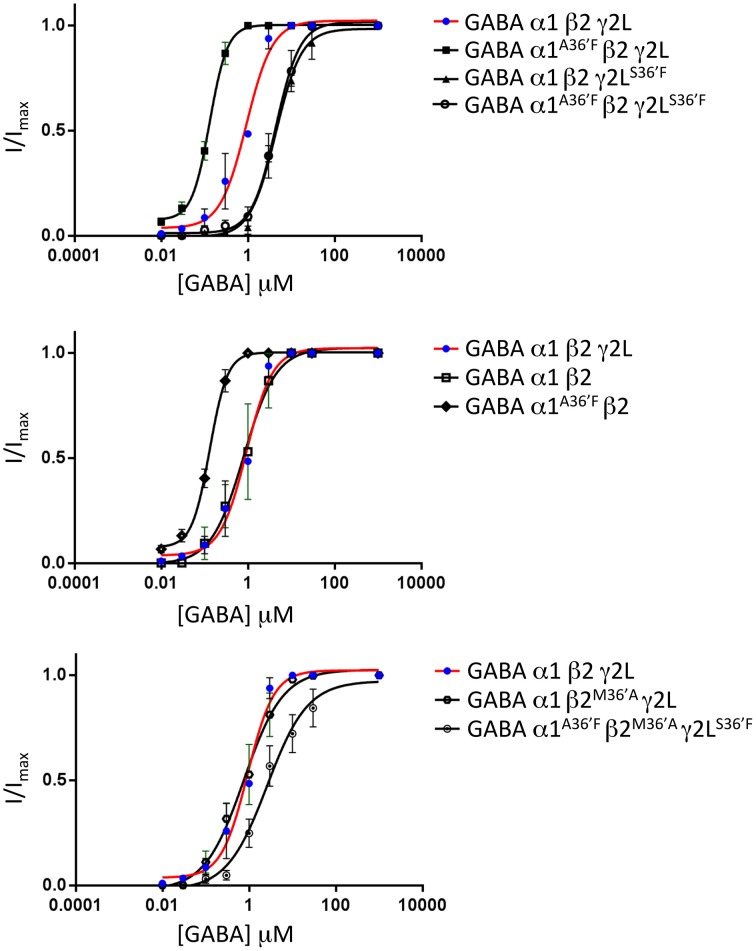
**Mean agonist concentration-response relationships of all GABA_A_R subunit combinations investigated in this study**. The wild type α1β2γ2L data, shown as blue points and red curve fits, are reproduced in all panels to facilitate comparison between panels. All data points were averaged from 4 to 6 cells and individual concentration-response curves were fitted by the Hill equation. The mean Hill equation parameters of best fit are summarized in Table 1.

The mean GABA concentration-response relationships for all wild type and mutant GABA_A_R subunit combinations investigated in this study are displayed in Figure 4, with mean parameters of best fit to the Hill equation summarized in Table 1. Of particular note, the α1^A36′F^ mutation caused a dramatic reduction in the GABA EC_50_ value whereas the γ2L^S36′F^ mutation had the opposite effect. This later effect provides strong evidence for the efficient incorporation of γ2L subunits into ternary receptors.

### Properties of α1^A36′F^β2γ2L GABA_A_Rs

**Figure 5:**
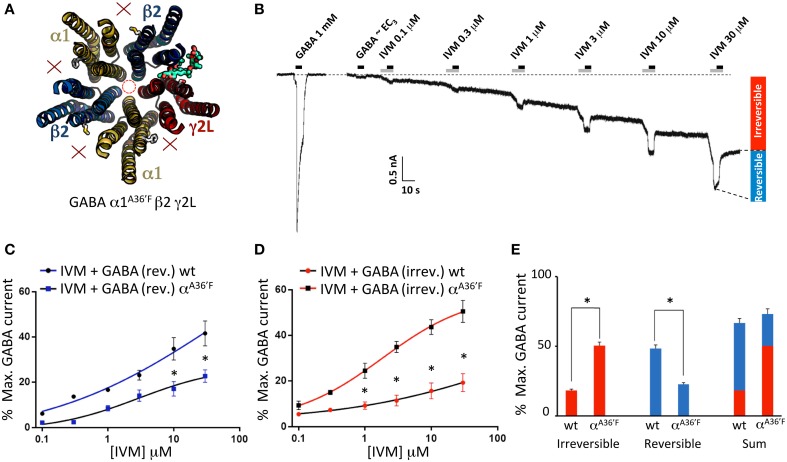
**Effects of ivermectin on α1^A36′F^β2γ2L GABA_A_Rs**. **(A)** Structural model showing the location of the single ivermectin binding site. **(B)** Sample recording showing the effect of increasing ivermectin concentrations on EC_3_ GABA-gated currents, with reversible (i.e., GABA-activated) and irreversible current increases indicated. **(C)** Mean concentration-response relationship of the reversible current component, compared with the corresponding wild type GABA_A_R data from Figure 1. **(D)** Mean concentration-response relationship of the irreversible current component, compared with the corresponding wild type GABA_A_R data from Figure 1. **(E)** Mean saturating magnitudes of reversible and irreversible ivermectin-modulated currents (defined as indicated in **B**), and their sum, plotted as a percentage of EC_3_ GABA-activated current, and compared to corresponding wild type data. ^*^Represents significance of *t*-test *P* < 0.05.

According to our molecular modeling, the α1^A36′F^ mutation blocks ivermectin binding at the α1-β and α1-γ2L interfaces, leaving the γ2L-β2 interface as the only available ivermectin site (Figure 5A). A sample recording, showing the effect of increasing ivermectin concentrations on EC_3_ GABA-gated currents on α1^A36′F^β2γ2L GABA_A_Rs, reveals both reversible (i.e., potentiating GABA-activated currents) and irreversible current increases (Figure 5B). The mean concentration-response relationship for the reversible current revealed no change in ivermectin sensitivity relative to the wild type receptor, although the saturating magnitude of the potentiation was significantly reduced (Figure 5C). The mean concentration-response of the irreversible current revealed a significantly larger peak current relative to that observed the wild type receptor (Figure 5D). Plotting the saturating reversible and irreversible current magnitudes as a percentage of the mean saturating GABA current supported these findings (Figure 5E). The main result is a significant increase in the ivermectin-induced irreversible current component in α1^A36′F^β2γ2L GABA_A_Rs relative to wild type GABA_A_Rs (Figure 5E).

### Properties of α1β2γ2L^*S*36′*F*^ and α1^A36′F^β2γ2L^*S*36′*F*^ GABA_A_Rs

**Figure 6:**
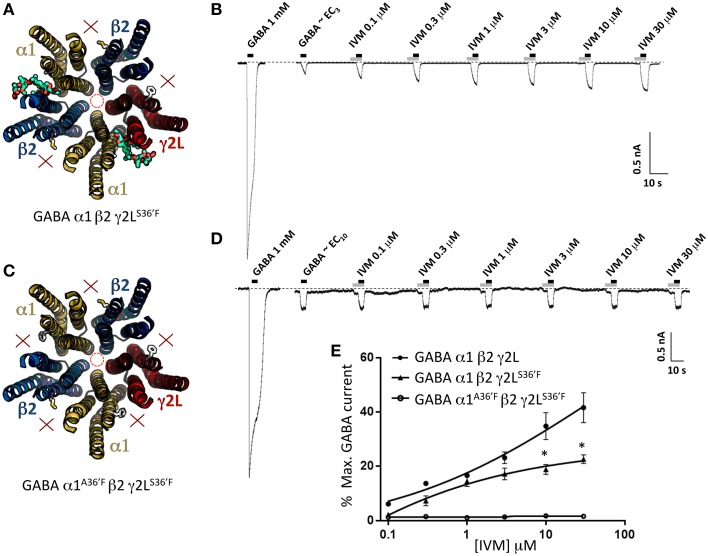
**Effects of ivermectin on α1β2γ2L^S36′F^ and α1^A36′F^β2γ2L^S36′F^ GABA_A_Rs**. **(A)** Structural model of α1β2γ2L^S36′F^ showing the location of the ivermectin binding sites. **(B)** Sample recording showing the effect of increasing ivermectin concentrations on EC_3_ GABA-gated currents in α1 β2γ2L^S36′F^ GABA_A_Rs. **(C)** Structural model of α1^A36′F^β2γ2L^S36′F^ showing the lack of ivermectin binding sites. **(D)** Sample recording showing the effect of increasing ivermectin concentrations on EC_10_ GABA-gated currents in α1^A36′F^β2γ2L^S36′F^ GABA_A_Rs. **(E)** Mean concentration-response data for the experiments as shown in **(B,D)**. ^*^Represents significance of *t*-test *P* < 0.05.

According to our modeling, α1β2γ2L^S36′F^ GABA_A_Rs should contain ivermectin sites at the α1-β2 and α1-γ2L interfaces only (Figure 6A). As shown in Figure 6B, ivermectin enhanced GABA-gated currents but induced no irreversible activation of these GABA_A_Rs. The averaged concentration-response relationship of the reversible potentiation revealed a reduction in its peak magnitude relative to the wild type receptor (Figure 6E), but no significant difference to that observed at the α1^A36′F^β2γ2L GABA_A_R (*P*>0.05, unpaired *t*-test). This similarity in potentiating efficacy may mean that the γ2L-β2 interface is functionally equivalent to the α1-β2 and α1-γ2L sites combined. However, the results may also be explained by the α1^A36′F^ mutation being ineffective in preventing ivermectin binding to the α1-β2 or α1-γ2L sites. We investigated this possibility in two-ways. First, we expressed α1^A36′F^β2γ2L^S36′F^ GABA_A_Rs which should contain no functional ivermectin sites (Figure 6C). If, on the other hand, the α1-β2 or α1-γ2L sites retain some residual functionality then this receptor should exhibit detectable ivermectin sensitivity. However, as shown in Figures 6D,E, no effect of ivermectin was observed at any concentration at α1^A36′F^β2γ2L^S36′F^ GABA_A_Rs.

### Properties of binary α1β2 GABA_A_Rs

**Figure 7:**
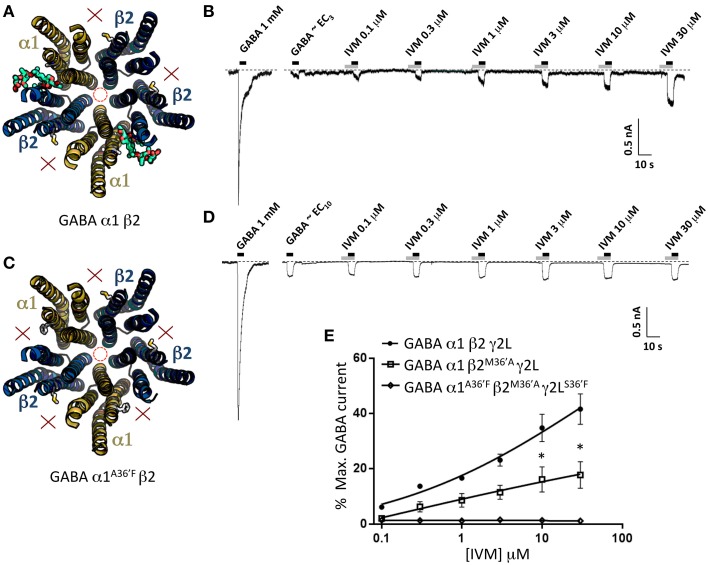
**Effects of ivermectin on α1β2 and α1^A36′F^β2 GABA_A_Rs**. **(A)** Structural model of α1β2 showing the location of the ivermectin binding sites. **(B)** Sample recording showing the effect of increasing ivermectin concentrations on EC_10_ GABA-gated currents in α1β2 GABA_A_Rs. **(C)** Structural model of α1^A36′F^β2 showing the lack of ivermectin binding sites. **(D)** Sample recording showing the effect of increasing ivermectin concentrations on EC_10_ GABA-gated currents α1^A36′F^β2 GABA_A_Rs. **(E)** Mean concentration-response data for the experiments as shown in **(B,D)**. ^*^Represents significance of *t*-test *P* < 0.05.

The second control experiment was to investigate the binary α1β2 GABA_A_R which exists in an obligatory α1-β2-α1-β2-β2 stoichiometry (Baumann et al., 2001). This receptor should thus have two functional α1-β2 ivermectin sites per pentamer (Figure 7A). As indicated in the sample recording in Figure 7B and in the averaged data plotted below, these receptors exhibited significant ivermectin-mediated potentiation of GABA-gated currents with a concentration-response profile indistinguishable from that of α1β2γ2L^S36′F^ GABA_A_Rs, but exhibited no irreversible ivermectin activation. As a negative control for this experiment we also tested α1^A36′F^β2 GABA_A_Rs, which like α1^A36′F^β2γ2L^S36′F^ GABA_A_Rs, should contain no functional ivermectin sites (Figure 7C). As predicted, this receptor exhibited no ivermectin sensitivity at all, even at concentrations up to 30 μM (Figures 7D, E). Taken together, results from Figures 6, 7 indicate that the α1^A36′F^ mutation completely eliminates ivermectin binding at the α1-β2 and α1-γ2L interface sites. Thus, we conclude that the maximum efficacy with which ivermectin potentiates GABA-gated currents is similar for receptors incorporating a single γ2L-β2 interface site as it is in receptors incorporating α1-β2 and α1-γ2L interface sites combined. We also conclude that irreversible ivermectin activation requires a functional γ2L-β2 interface site.

### Properties of GABA_A_Rs incorporating mutant β2-M36′A subunits

**Figure 8:**
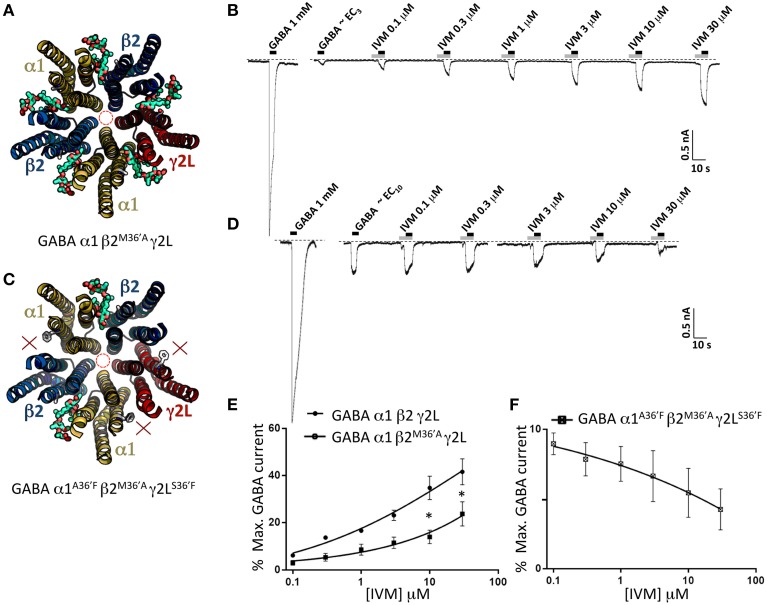
**Effects of ivermectin on α1β2^M36′A^γ2L and α1^A36′F^β2^M36′A^γ2L^S36′F^ GABA_A_Rs**. **(A)** Structural model of α1β2^M36′A^γ2L showing the location of the ivermectin binding sites. **(B)** Sample recording showing the effect of increasing ivermectin concentrations on EC_3_ GABA-gated currents in α1β2^M36′A^γ2L GABA_A_Rs. **(C)** Structural model of α1^A36′F^β2^M36′A^γ2L^S36′F^ showing the location of the single ivermectin binding site. **(D)** Sample recording showing the inhibitory effect of increasing ivermectin concentrations on EC_10_ GABA-gated currents in α1^A36′F^β2^M36′A^γ2L^S36′F^ GABA_A_Rs. **(E)** Mean concentration-response data for the experiments as shown in **(B)**. **(F)** Mean concentration-response data for the experiments as shown in **(D)**. ^*^Represents significance of *t*-test *P* < 0.05.

To help validate the role of 36′ sidechains in forming ivermectin sites, we investigated the functional properties of GABA_A_Rs incorporating the β2^M36′A^ mutation. We first characterized the ivermectin sensitivity of the α1β2^M36′A^γ2L GABA_A_R which should incorporate five ivermectin sites per pentamer (Figure 8A). The effects of increasing ivermectin concentrations on EC_3_ GABA-gated currents in this receptor revealed significant potentiation of GABA-gated currents but no irreversible activation (Figure 8B). The averaged ivermectin concentration-response relationship demonstrates that the magnitude of the reversible potentiation to be significantly smaller than that observed at the wild type receptor (Figure 8E) although it was not significantly different to that observed at α1β2γ2L^S36′F^ or α1^A36′F^β2γ2L GABA_A_Rs (*P*>0.05 unpaired *t*-tests). This result was unexpected for two reasons. First, despite a presumably intact γ2L-β2 binding site, it exhibited no irreversible ivermectin activation, apparently contradicting our conclusion from the previous section. Second, it seems surprising that receptors with five ivermectin sites should yield a reduced ivermectin efficacy relative to wild type receptors which have only three sites. We then investigated the α1^A36′F^β2^M36′A^γ2L^S36^′ GABA_A_R, which should incorporate ivermectin sites at the two β2-α1 interfaces only (Figure 8C). As shown in the sample recording (Figure 8D) and in the averaged concentration-response data (Figure 8E), EC_10_ GABA-gated currents were strongly inhibited by ivermectin. This unexpected result provides a possible explanation as to why the ivermectin potentiating and direct activation efficacy at α1β2^M36′A^γ2L GABA_A_Rs was reduced relative to wild type GABA_A_Rs.

### Quantitating the magnitude of ivermectin-activated currents by picrotoxin block

**Figure 9:**
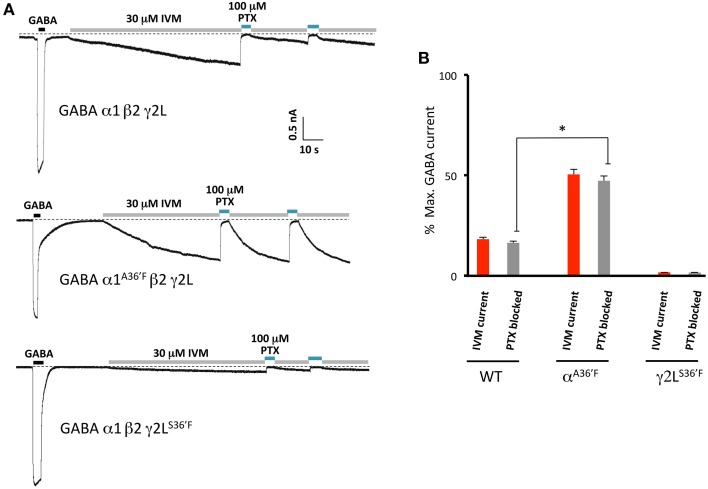
**Picrotoxin block of currents directly activated by ivermectin**. **(A)** Examples of ivermectin activation and picrotoxin block of GABA_A_Rs comprising the indicated subunits. **(B)** Averaged picrotoxin block data from **(A)** compared with averaged ivermectin irreversible activation data from Figure 5E. All results were averaged from at least 4 cells. This confirms that direct ivermectin activation requires receptors with intact γ2L-β2 interfaces. ^*^Represents significance of *t*-test *P* < 0.05.

In a final set of experiments, we sought to confirm the relative magnitudes of the irreversible ivermectin-gated currents in α1β2γ2L, α1^A36′F^β2γ2L, and α1β2γ2L^S36′F^ GABA_A_Rs using picrotoxin block. Our approach involved applying 30 μM ivermectin until maximal activation was achieved, and then blocking this current with 100 μM picrotoxin (Figure 9A). The mean picrotoxin-blocked current was then expressed as a percentage of the saturating GABA-activated current, and compared to the irreversible ivermectin-activated current magnitude replotted from Figure 5E. The averaged results (Figure 9B), confirm that ivermectin activates irreversible currents in the α1β2γ2L and α1^A36′F^β2γ2L GABA_A_Rs but not in the α1β2γ2L^S36′F^ GABA_A_R.

## Discussion

### Validation of mutagenesis approach

An overarching hypothesis of this study is that the ability of ivermectin to bind to a specific interface is determined by the sidechain volume of 36′ residue on the + side of that interface. We have shown using binary α1^A36′F^β2 receptors that the α1^A36′F^ mutation eliminates ivermectin sensitivity at α1-β2 interfaces. We have also shown, via the ternary α1^A36′F^β2γ2L^S36′F^ receptor, that 36′F mutations eliminate ivermectin sensitivity at the α1-γ2L and γ2L-β2 interfaces. We also performed the reverse control experiment whereby we introduced the β2^M36′A^ mutation into ivermectin-insensitive α1^A36′F^β2γ2L^S36′F^ receptors to create ivermectin sensitivity where previously there was none (Figure 8E). Hence, as with GlyRs and GluClRs (Lynagh and Lynch, 2010), a bulky 36′F sidechain eliminates ivermectin binding, whereas a small sidechain (36′G, 36′A, or 36′S) is conducive to ivermectin binding. Thus, 36′ sidechain volume is a useful means of controlling ivermectin binding to particular interfaces in GABA_A_Rs.

### Main findings

Our first main finding concerns the relationship between the efficacy with which ivermectin potentiates EC_3_ GABA-gated currents and the number of functional ivermectin sites per receptor. Wild type α1β2γ2L GABA_A_Rs, which show greatest efficacy, incorporate three ivermectin sites. GABA_A_Rs that incorporate two native ivermectin sites (i.e., the α1β2γ2L^S36′F^ and α1β2 receptors) show a correspondingly reduced level of ivermectin efficacy. However, the α1^A36′F^β2γ2L GABA_A_R, which contains a single ivermectin site at the γ2L-β2 interface, has an efficacy similar to that of receptors containing two sites. This suggests the γ2L-β2 interface site is more allosterically active. One possibility is that bound ivermectin induces a large conformational and/or energetic change here that perhaps also locks ivermectin into the pocket thereby causing irreversible activation. In support to this hypothesis, our docking analysis shows more residues interacting with ivermectin on the γ2L (+) side in comparison with the α1 (+) side, which should enhance the stability of ivermectin binding (Figure 2C).

Our second main finding is that the irreversible activating effect of ivermectin requires a functional ivermectin site at the γ2L-β2 interface. Is there a molecular explanation for this apparently strong binding interaction? In their GluClR-ivermectin crystal structure, Hibbs and Gouaux originally concluded that ivermectin bound via H-bonds with Q-26′, S15′, and T40′ and Van der Waals interactions with several other residues (Hibbs and Gouaux, 2011). Of the H-bonding residues, α1 and γ2L subunits both contain Q-26′ and S15′ residues, and consequently they are unlikely to specifically mediate the differential effect. On the other hand, α1 has a 40′A whereas γ2L has a 40′I, although neither of these residues contribute to H-bonds. In any case, the importance of any putative H-bonding by 40′ residues has been questioned (Lynagh and Lynch, 2012a). The irreversible binding effect of ivermectin in the γ2L-β2 interface site is thus more likely to be due to the additional weak interactions in the binding pocket, rather than to the presence or absence of specific strong binding interactions. This fits well with our modeling results (Figure 3) which propose that the γ2L-β2 interface shows a greater number of residues contributing polar contacts which should tighten the pocket around the ivermectin molecule.

If ivermectin binds irreversibly via the γ2L-β2 interface site as we conclude above, then why does the α1β2^M36′A^γ2L GABA_A_R exhibit no detectable irreversible ivermectin activation? We suggest this might be due to the presence of the decrypted ivermectin site at the β2^M36′A^-α1 interface. This site, which in isolation mediates ivermectin inhibition of GABA-gated currents (Figure 8E), may either exert a closing effect on the channel to counter the irreversible activation, or it might allosterically interact with the γ2L-β2 site to render it non-functional. These possibilities would be difficult to separate experimentally. The observed reduction in the efficacy of ivermectin potentiation of reversible EC_3_ GABA-gated currents at the α1β2^M36′A^γ2L GABA_A_R might be explained similarly (Figure 8E). An alternate possibility is that the mutant β2^M36′A^ subunit impairs expression of the γ2L subunit, which would also eliminate the irreversible ivermectin binding site.

Our third main finding is that ivermectin inhibits GABA-gated currents by binding to decrypted β2^M36′A^-α1 interface binding sites in the α1^A36′F^β2^M36′A^γ2L^S36′F^ GABA_A_R. As these sites are lined by the M2 and M3 domains from the β2 subunit and the M1 domain from the α1 subunit, M1 residues that are not conserved between α1 and β2 or γ2, or M2/3 residues that are not conserved between β2 and α1 or γ2, could be key to forming inhibitory ivermectin sites. These criteria are satisfied by two putative ivermectin H-bonding residues (β2^15′N^ and β2^40′V^) plus several other residues that contribute weak interactions to bound ivermectin (α1^−19′M^, α1^−22′P^, α1^−23′L^, α1^−27′I^, and β2^33′M^). According to our modeling, all but one of these residues contacts ivermectin (Figure 3). As other non-conserved residues in the TM domains could also be important in terms of altering the overall shape of the pocket, the molecular elements essential for conferring the inhibitory action of ivermectin at the β2^M36′A^-α1 interface may be difficult to isolate.

Inhibitory effects of ivermectin have previously been characterized in other pLGICs. For example, in the α1 GlyR it was shown that bulky tryptophan substitutions to residues midway down the M1 domain (L-19′W) or M3 domain (L39′W) convert ivermectin from an agonist into an inhibitor of glycine-activated currents (Lynagh et al., 2011). Similar effects have been observed in α7 nAChRs incorporating mutations to residues in the M1, M2, or M3 domains (S-17′M, M15′L, and S37′V, respectively; Collins and Millar, 2010). In the *Drosophila* RDL pLGIC, ivermectin was converted into an inhibitor by a variety of single mutations including A2′C, A2′S, A2′N, G34′T, V38′Q, and V38′N (Nakao et al., 2015). Taken together, the broad distribution of these residues provides few clues as to specific elements responsible for ivermectin inhibition at the β2^M36′A^-α1 interface. Again, the inhibitory effect of ivermectin could be due to the overall shape of the binding pocket, and the nature of the conformational change that occurs when it is occupied.

### Binding of other molecules in and near the ivermectin site

The 36′ position has been implicated in alcohol and anesthetic specific modulation (Mascia et al., 2000), as well as neurosteroids (Akk et al., 2007; Li et al., 2009) and even cholesterol (Hénin et al., 2014). Ivermectin “wedges” between the M3 helix of the principal subunit and the M1 helix of the complimentary subunit of the GluClR to stabilize the wide-open pore conformation (Hibbs and Gouaux, 2011), and the access to this inter-membrane binding pocket is granted (or not) by the residue occupying the M3 36′ position. Ivermectin removal was first predicted to shrink the GluClR pore radius to 1.2 Å (Yoluk et al., 2013). Subsequent publication of the GluClR crystal structure in the apo-conformation not only supported this model but also showed that other hydrophobic molecules including membrane phospholipids are able to interact with this pocket to modulate the pore (Althoff et al., 2014). If this pocket is accessible to phospholipids, endogenous neurosteroids or cholesterol, then ivermectin may be able to compete with and displace some of these molecules with a potency that may vary from one interface to the next.

The cooperative effect between transmembrane interfaces has previously been showed in the human α1β3γ2 GABA_A_R by the use of a potent stereospecific photoreactive modified barbiturate in combination with the anesthetic etomidate (Chiara et al., 2013). The barbiturate did not photolabel the etomidate-binding site located at the β3-α1 interface, but instead it did so at the α1-β3 and γ2-β3 interfaces involving, among others, some of the residues that contribute to ivermectin sensitivity. Each ligand (etomidate and the photoreactive barbiturate) could enhance the photo-incorporation of the other, demonstrating allosteric interactions between sites, and potentiating their effect in combination. This mechanism was hypothesized to bypass the extracellular domain-transmembrane domain allosteric coupling regulated by the binding of the neurotransmitter to its orthosteric-binding site (Sauguet et al., 2015). The results we present here may also be interpreted in the same manner.

## Conclusion

We show that ivermectin binds to α1-β2, α1-γ2L, and γ2L-β2 subunit interface sites in α1β2γ2L GABA_A_Rs. These binding sites are not equivalent. For example, ivermectin produces irreversible channel activation only when it binds to the γ2L-β2 interface site. When it binds to α1-β2 sites it elicits potentiation of GABA-gated currents but has no irreversible activating effect. Ivermectin cannot bind in the β2-α1 interface binding site due to a bulky methionine sidechain at the M3 36′ position that blocks access to the ivermectin pocket. Introducing a small alanine residue at this interface (M36′A) creates a functional ivermectin site, but surprisingly it is inhibitory with bound ivermectin reducing the magnitude of GABA-gated currents. Our molecular modeling simulations predict that ivermectin binds in a common orientation at each interface, although it is coordinated by a larger number of molecular interactions at the γ2L-β2 interface site than at any other interface. This may explain why ivermectin binds irreversibly here. Overall, this study demonstrates unexpectedly stark pharmacological differences among the ivermectin binding sites in GABA_A_Rs that cannot easily be explained by non-conserved residues in or around these sites. Rather, these results provide evidence that the ivermectin sites have subtly different structures and occupation of them by ivermectin induces subtly different conformational changes that modulate channel function in different ways. Understanding these structural and conformational subtleties could provide insights into the development of new drugs for a variety of neurological conditions.

## Author contributions

AE-M conducted all experiments, analyzed the data and wrote the manuscript. JL designed the project, analyzed the data and wrote the manuscript.

## Funding

This project was supported by the Australian Research Council (LP120100297, DP120104373) and the National Health and Medical Research Council of Australia (1058542, 1060707). AE was supported by a University of Queensland Postdoctoral Research Fellowship.

### Conflict of interest statement

The authors declare that the research was conducted in the absence of any commercial or financial relationships that could be construed as a potential conflict of interest.
